# Compatibility Study of Danggui Buxue Tang on Chemical Ingredients, Angiogenesis and Endothelial Function

**DOI:** 10.1038/srep45111

**Published:** 2017-03-22

**Authors:** Ping-Lan Lin, Zhi-Cheng Li, Rui-Fang Xie, You-Hua Wang, Xin Zhou

**Affiliations:** 1 Department of Pharmacy, Longhua Hospital Affiliated to Shanghai University of Traditional Chinese Medicine, Shanghai, China; 2 Department of nephrology, Shuguang Hospital Affiliated to Shanghai University of Traditional Chinese Medicine, Shanghai, China; 3 Shanghai Pu Dong Hospital affiliated to FuDan University, Shanghai, China; 4 Hypertension lab, Cardiovascular Department, Longhua Hospital Affiliated to Shanghai University of Traditional Chinese Medicine, Shanghai, China

## Abstract

Danggui Buxue Tang (DBT) is a classic Chinese herbal formula which consists of *Astragali mongholici Radix* and *Angelica sinensis Radix* (ASR). For chemical ingredients, HPLC were performed. Results showed compared with single herbs, DBT decoction could promote the dissolution of ingredients such as ferulic acid and calycosin. Furthermore, when ratio of AMR to ASR was 5 to 1, synthetic score was the best. For angiogenesis, normal and injured zebrafish model were applied. Results showed DBT and its ingredients had angiogenesis effects on Sub Intestinal vessels (SIVs) of normal zebrafish. Meanwhile, DBT and its single herbs could also recover Inter-Segmental Vessels (ISVs) injured by VRI. Angiogenesis effects of DBT on ISVs were better than single herbs. AMR extract, Total Saponins of AMR, Polysaccharide of ASR, ferulic acid, calycosin and calycosin-7-glucoside could be effective ingredients for angiogenisis. For endothelium functions, Lysoph-Osphatidyl choline was used to damage rat endothelial function of thoracic aorta. The results showed DBT and its single herbs could improve endothelial dysfunctions in dose-dependence. Both ferulic acid and calycosin-7-glucoside could also improve endothelium dysfunction in dose dependence. Therefore, compatibility of DBT was reasonable. Compared with single herbs, DBT could promote dissolution of effective ingredients, enhance angiogenesis and relieve endothelial dysfunction.

Danggui Buxue Tang (DBT) was first described in the Treatise in Dong-Han in 1247 AD. It is a classic prescription which has been used for “regulating and enriching blood” for thousands years. It consists of *Astragali mongholici Radix* (AMR) and *Angelica sinensis Radix* (ASR).

According to Traditional Chinese Medicine (TCM) theory, AMR can treat “qi” deficiency while ASR can replenish the blood. “Qi” (vital energy) and “Blood” (materials) are considered to be closely related. Thus, the “Qi” tonified by AMR can enhance “Blood” functions promoted by ASR. Further modern pharmacological researches have confirmed that DBT had hematopoietic^1^ and angiogenic effects^2^. It could also regulate immune system^3^, protect cardiovascular system^4^ and participate in blood lipid regulation^5^. Therefore, currently DBT is used to treat cardiovascular diseases in clinical practice^6^.

In DBT, the ratio of AMR to ASR is usually reported as 5:1^7^. Beside this ratio (5:1), other ratios were also documented in literatures of TCM with ratios of 5:6, 2:1, 10:3, 4:1 and 5:2^8^ (AMR:ASR).

How does DBT produce therapeutic effects for cardiovascular diseases? What are possible mechanisms? Whether will different proportions of DBT ingredients impact on its effects? In order to answer these questions, High Performance Liquid Chromatography (HPLC), Tg (Fli1: EGFP) transgenic zebrafish model and rat endothelial dysfunction model were adopted to investigate the active ingredients and pharmacological functions of DBT in present study.

## Results

### Quality control of herbs in DBT

**Figure 1:**
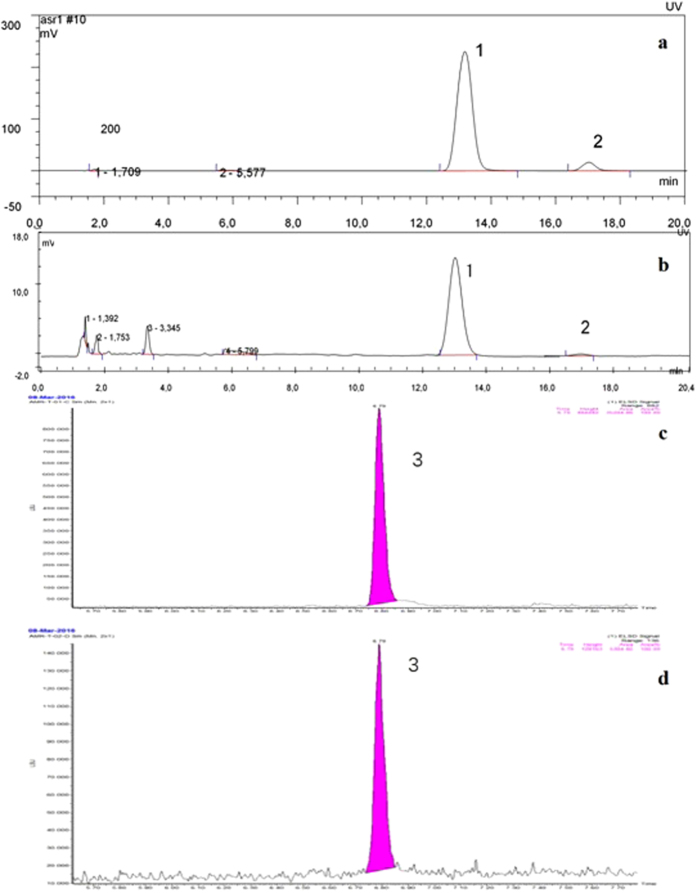
HPLC chromatograms of FA and cis-FA reference solution (**a**), ASR (**b**), astragaloside IV (**c**) and AMR (**d**). peak 1:trans-ferulic acid; peak 2:cis-trans ferulic acid; peak 3: astragaloside IV.

The chromatographic peaks of ferulic acid in the herbal extract of ASR and astragaloside IV in the powder of AMR, as well as the peak of reference solution were shown in Fig. 1a,b and c,d, respectively. The retention time of trans-ferulic acid was about 13.5 min, cis-ferulic acid was about 17.2 min. The system suitability of the two peaks in the chromatograph of reference solution was calculated as 6.3, which was satisfied with the monograph (minimum 1.3). The content in dried drug was 0.078%, which was consisted within the criterion (minimum 0.050%). As meanwhile, the retention time of astragaloside IV was about 6.75 min. The system suitability of the two peaks in the chromatograph of reference solution was calculated as 6.3, satisfing with the monograph (minimum 4.0). The content in dried drug was 0.19%, meeting the requirement (minimum 0.04%).

### Contents of chemical ingredients in DBT

**Figure 2:**
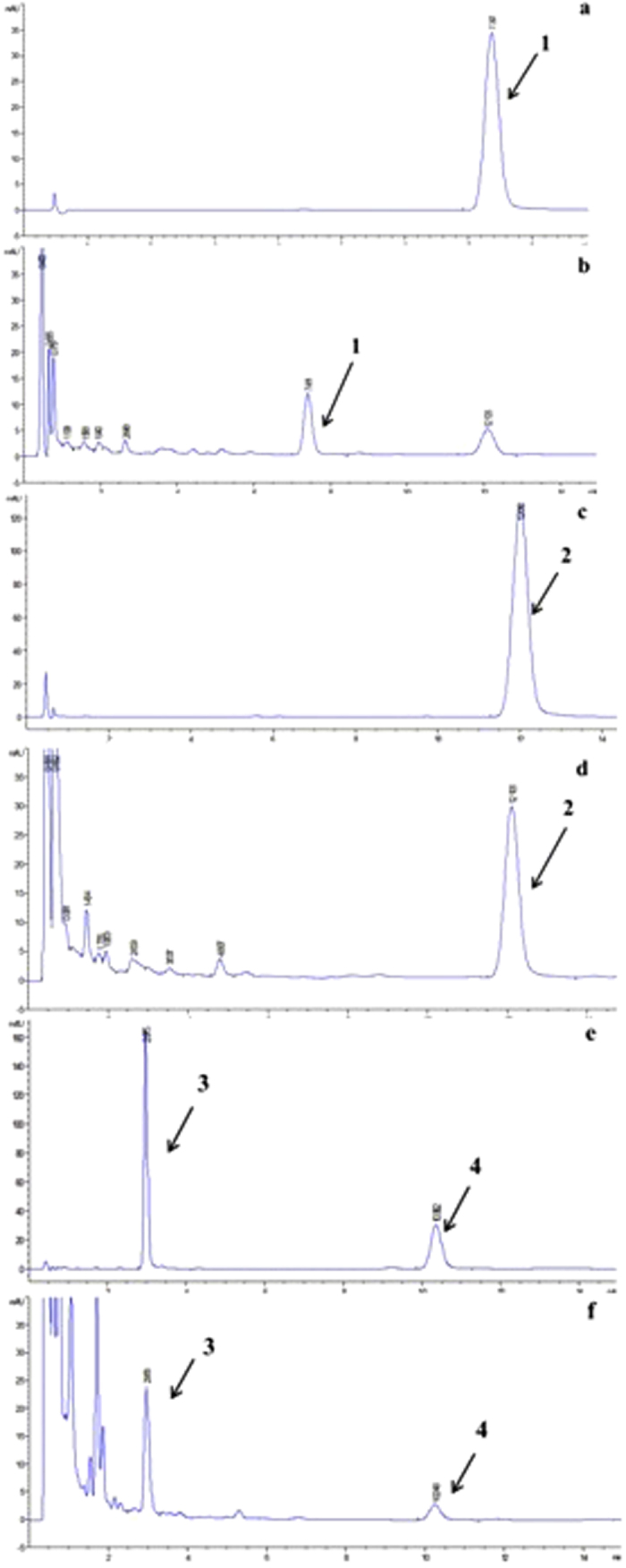
HPLC chromatograms of FA(**a**), calycosin-7-glucoside (**c**), calycosin (**e**), formononetin (**e**) and DBT(**b**, **d**, **f**). Peak 1: FA; peak 2: calycosin-7-glucoside; peak 3: calycosin; peak 4: formononetin.

To determine the contents of chemical ingredients in DBT, a HPLC method was established, and the majority of peaks showed satisfactory baseline separation (Fig. 2).

Based on the developed method, a good linearity of each marker ingredient was observed in a relatively wide concentration with the correlation coefficient larger than 0.995 (Table 1). In the recovery assay, four compounds showed mean recovery rates in the range of between 90% and 110%, indicating an acceptable accuracy of the method (Table 2). Limits of Detection (LOD) and Limits Of Quantification (LQD) were also listed in Table 2. Both reproducibility and stability were also satisfactory, with Relative Standard Deviations (RSDs) less than 2% (Tables 3 and 4). In general, the proposed method exhibited a satisfactory linearity, reliable accuracy and good precision and could be used for further analysis of the DBT samples.

The quantification results showed that contents of ferulic acid and calycosin-7-glucoside in DBT were obviously higher than single herbs (Table 5) while the contents of calycosin and formononetin in DBT were less than those in AMR, indicating that formula decoction could promote the dissolution of water soluble ingredients. Furthermore, when the ratio of AMR to ASR was respectively 5 to 1 and 4 to 1, the synthetic scores (Table 6) was the highest, indicating the classic ratio of formula (5:1) was favorable from the view of chemical ingredients.

### Evaluation of drug toxicity on zebrafish

The toxicities of different drugs on zebrafish were shown in Table 7. The maximum nontoxic concentrations of the herbal decoction were 250 μg/ml; The maximum nontoxic concentrations of extract of herbs, including AMR extract, Total Saponins of AMR(AMRTS), Polysaccharide of AMR (AMRP), Polysaccharide of *Angelica sinensis* Radix (ASRP) and ASR extract were 100 μg/ml; The maximum nontoxic concentrations of active compounds in herbs, including calycosin, calycosin-7-glucoside, formononetin, astragaloside IV and ferulic Acid, were between 10 to 100 μM. Further experiment was performed using concentrations lower than the maximum nontoxic concentrations.

### Effects of different drugs on SIVs of normal zebrafish

**Figure 3:**
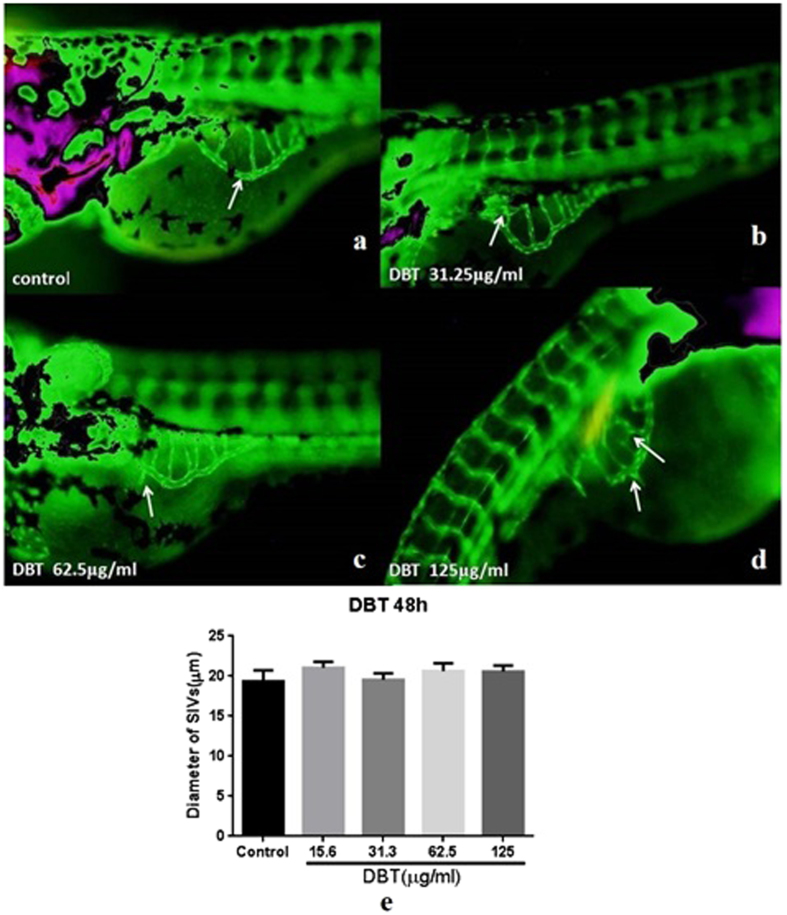
Effects of DBT on the diameters of SIVs of Tg (fli1: EGFP) zebrafish (n = 6). Embryos were separately treated with control medium (**a**), 31.25 μg/ml (**b**), 62.5 μg/ml (**c**), 125 μg/ml (**d**) DBT for 48 h. The diameters of SIV were quantitatively measured (**e**).

In experiments conducted on normal zebrafish, the Sub-Intestinal vessels (SIV) area of control group was a smooth arch net structure inside with 4~6 vessels (Fig. 3a). After incubation with different concentrations of DBT for 48 h, the buds appeared (Fig. 3b,c,d) and diameters of SIVs increased (Fig. 3e).

**Figure 4:**
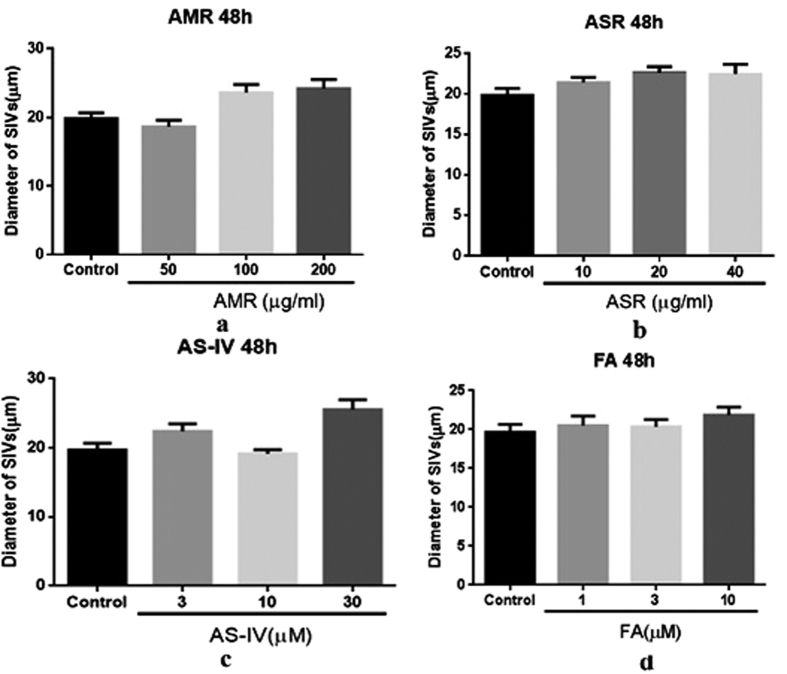
Effects ofAMR, ASR and pure compounds on the diameters of SIVs of Tg(fli1:EGFP) zebrafish (n = 6). Embryos were separately treated with AMR, ASR, AS-IV and FA at different concentrations for 48 h. The diameters of SIV of the zebrafish treated with AMR (**a**), ASR (**b**), AS-IV (**c**) and FA (**d**) were measured.

In addition, the effects of the constitutive herbs and active compounds of DBT on the diameters of SIV were also investigated. Our results showed that the herbs and ingredients promoted the growth of SIVs diameters (Fig. 4). Furthermore, the effects of AMR (Fig. 4a) and Astragaloside IV (AS-IV) (Fig. 4c) were better than those of ASR (Fig. 4b) and FA (Fig. 4d). The results indicated that DBT and its constitutive herbs could promote SIVs growth in normal zebrafish and the effects might be attributable to AS-IV and FA.

### Effects of drugs on damaged zebrafish

#### Effects of DBT and its single herbs

**Figure 5:**
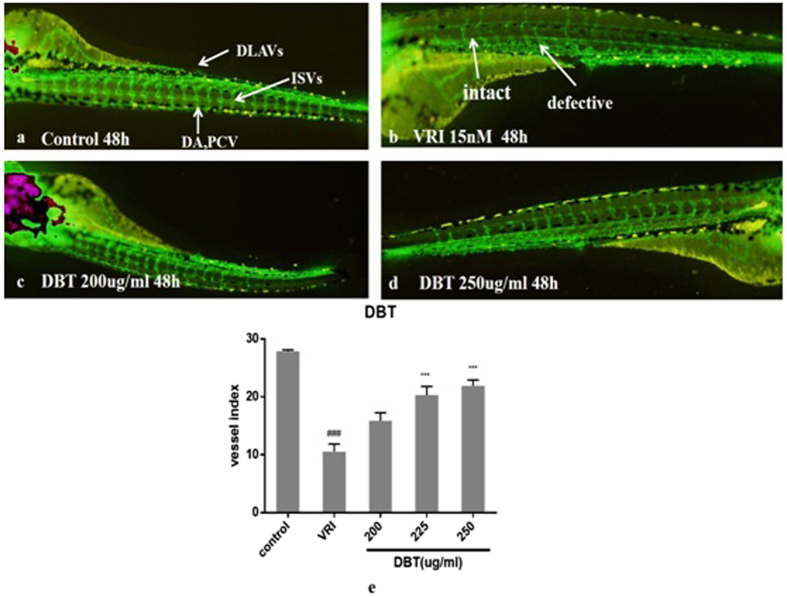
Effects ofDBTon ISVs of Tg(fli:EGFP) zebrafish after treatment with VRI (n = 6). Embryos were treated without (**a**) or with VRI for 3 h at 15 nM(**b**,**c**,**d**). After washing three times with medium, the embryos were treated with DBT at 200 μg/ml (**c**), 225 μg/ml and 250 μg/ml (**d**). The numbers of ISVs were recorded at 48 h(**e**). ^#^*p* < 0.05, ^##^*p* < 0.01, ^###^*p* < 0.001 when compared with the vehicle group; *p < 0.05, **p < 0.01, ***p < 0.001 when compared with the model group.

To study the angiogenic effects of DBT and its constitutive herbs on injured vessels, VEGFR Tyrosine Kinase Inhibitor II(VRI) was used to inhibit Inter-Segmental Vessels (ISVs). In the normal group, ISVs connected with Dorsal Aorta (DA) and Dorsal Longitudinal Anastamotic Vessels (DLAVS) which were intact (Fig. 5a) and the average number of SIVs were 28 (Fig. 5e control). After incubation with VRI for 3 h, some ISVs were not connected DA or Posterior Cardinal Vein(PCV) to DLAVs (Fig. 5b), indicating the growth of ISVs was inhibited by VRI. The average number of intact ISVs in the model group was around 10 (Fig. 5e VRI).

When DBT was incubated with zebrafish at different concentrations for 48 h, the numbers of intact ISV significantly increased (Fig. 5c,d) in a dose-dependent manner (Fig. 5e), indicating that DBT had the angiogenic effects on injured ISVs.

**Figure 6:**
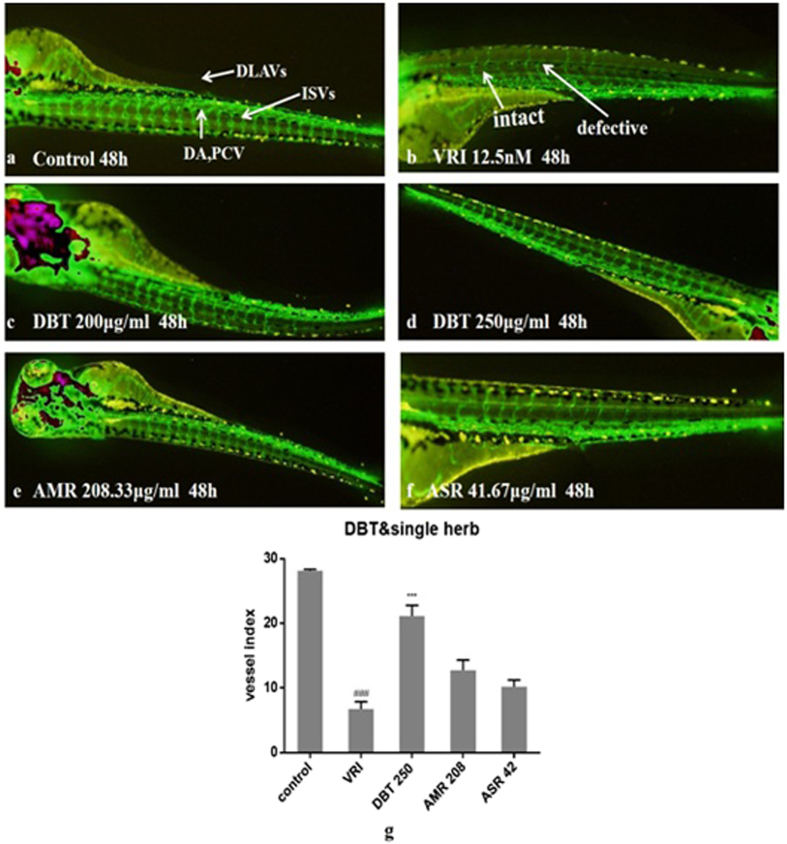
Effects of DBT, AMR and ASR on ISVs numbers of Tg(fli1:EGFP) zebrafish after treatment with VRI (n = 6). Embryos were treated without (**a**) or with VRI at 12.5 nM for 3 h (**b**,**c**,**d,e**,**f**). After washing three times with medium, the embryos were respectively treated with DBT at 200 μg/ml (**c**), 250 μg/ml (**d**), AMR at 208.33 μg/ml (**e**) and ASR at 41.67 g/ml (**f**). The numbers of ISVs were recorded at 48 h(**g**). ^#^*p* < 0.05, ^##^*p* < 0.01, ^###^*p* < 0.001 when compared with vehicle group; *p < 0.05, **p < 0.01, ***p < 0.001 when compared with the model group.

Further experiments also showed the ISV number of the DBT group (in which the ratio of AMR to ASR was 5 to 1) (Fig. 6d) were higher than those of the AMR and ASR groups (Fig. 6e,f), indicating that the formula produced better effects than single herbs(Fig. 6g).

**Figure 7:**
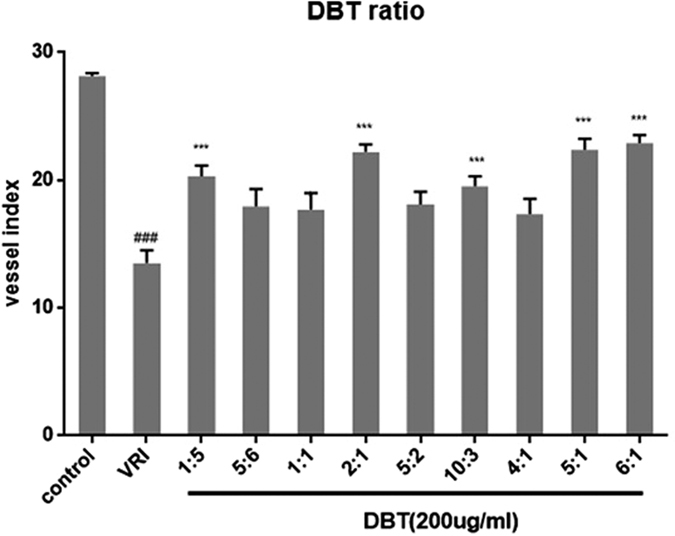
Effects of different ratios DBT on ISVs numbers of Tg(fli1:EGFP)zebrafish after inhibition with VRI (n = 6). Embryos were treated without or with VRI at 12.5 nM for 3 h. After washing three times with medium, the embryos were respectively with different ratiosof DBT. The numbers of ISVs were recorded at 48 h. Compared with vehicle group: ^#^*p* < 0.05, ^##^*p* < 0.01, ^###^*p* < 0.001; Compared with model group:*p < 0.05, **p < 0.01,***p < 0.001.

Effects of different proportions of DBT on ISVs were also investigated. The results showed that all DBT treatments could relieve the injuries of ISVs induced by VRI (Fig. 7). Different ratios of AMR to ASR (1:5, 2:1, 5:2, 5:1 and 6:1) resulted in significant disparity on relieving the effects of VRI. Among them, classic ratios of AMR to ASR (5:1) had the best effects.

#### Effects of AMR extract and its pure compounds

**Figure 8:**
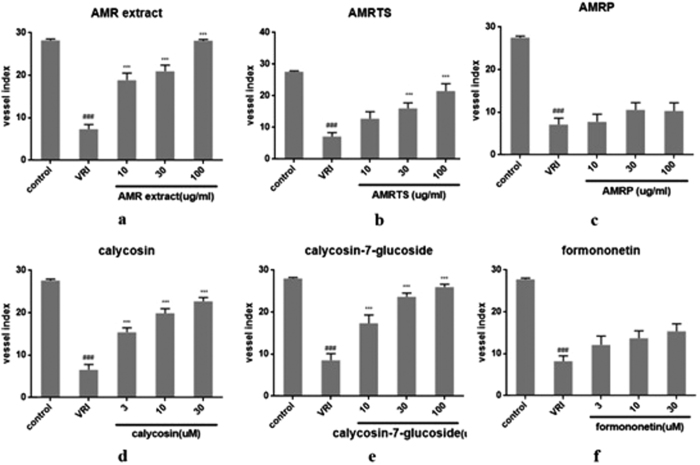
Effects of AMR extract, AMRTS, AMRP, calycosin, calycosin-7-glucoside, formononetin, astragaloside IV on the ISVs numbers of Tg(fli1:EGFP) zebrafish after treatment with VRI (n = 6). Embryos were treated without or with VRI at 15 nM for 3 h. After washing three times with medium, the embryos were respectively treated withAMRextract (**a**), AMRTS (**b**), AMRP (**c**), calycosin (**d**), calycosin-7-glucoside (**e**), formononetin (**f**) at different concentrations. The numbers of ISVs were recorded at 24 h and 48 h respectively. ^#^*p* < 0.05, ^##^*p* < 0.01, ^###^*p* < 0.001 when compared with vehicle group; *p < 0.05, **p < 0.01, ***p < 0.001 when compared with model group.

In the AMR total extract group, the numbers of ISVs were markedly higher than that of VRI groups at 48 h (Fig. 8a), and the difference increased in a dose-dependent manner. It has been known that AMR possesses various kinds of polysaccharide and saponins. Therefore, we investigated the angiogenic functions of its polysaccharides and total saponins. Results showed that the angiogenic effects of AMRTS on ISVs (Fig. 8b) were more potent than those of the polysaccharides of AMRP (Fig. 8c). Pure compounds including calycosin (Fig. 8d), calycosin-7-glucoside (Fig. 8e) and formononetin (Fig. 8f) exerted angiogenic effect, indicating that flavones had pro-angiogenisis functions.

#### Effects of ASR extract and its pure compound

**Figure 9:**
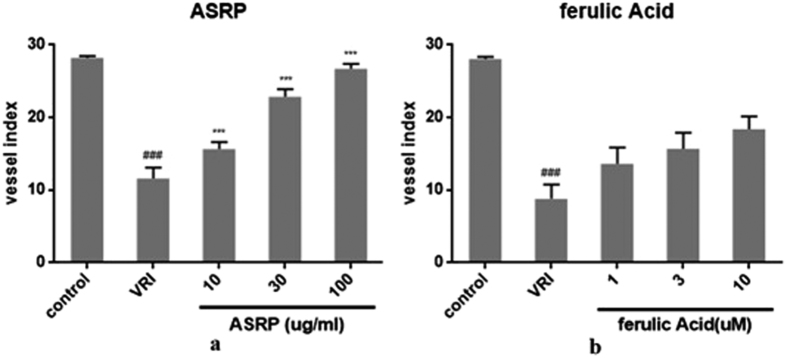
Effects ofASRP, FAon the ISVs numbers of Tg(fli1:EGFP) zebrafish after treatment with VRI (n = 6). Embryos were treated without or with VRI at 15 nM for 3 h. After washing three times with medium, the embryos were respectively treated withASRP (**a**) and FA (**b**)at different concentrations. The numbers of ISVs were recorded at 48 h respectively. ^#^*p* < 0.05, ^##^*p* < 0.01, ^###^*p* < 0.001 when compared with the vehicle group; *p < 0.05, **p < 0.01, ***p < 0.001 when compared with the model group.

ASRP was shown to have obvious angiogenic effect in a dose-dependent pattern (Fig. 9a). Meanwhile, pure compounds ferulic acid in ASR could also promote the growth of ISVs (Fig. 9b).

### Results of endothelial function

#### Establishment of endothelial dysfunction model

**Figure 10:**
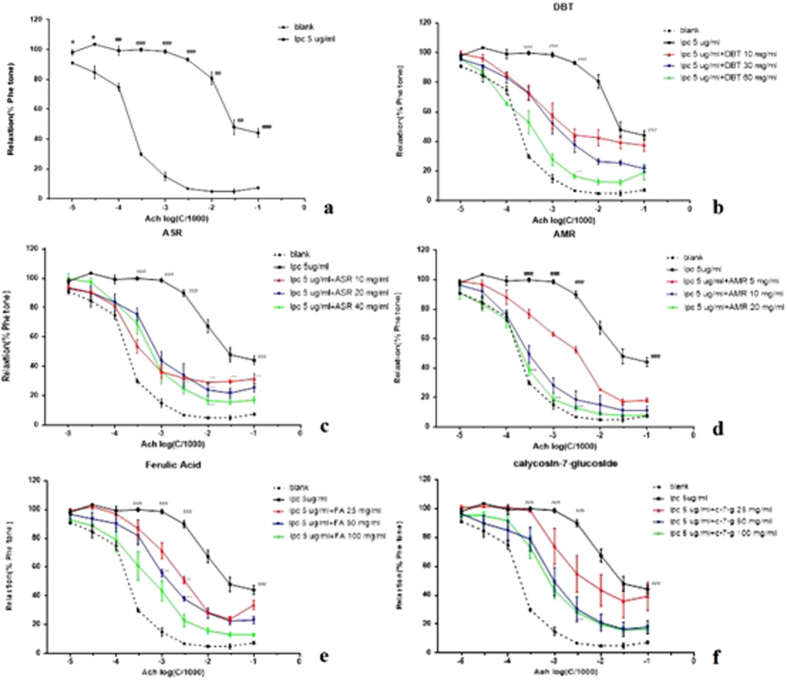
Influences of DBT(**b**), ASR (**c**), AMR (**d**), Ferulic acid (**e**) and Calycosin-7-glucoside(**f**) on endothelial dysfunction induced by LPC(n = 3). Rings were induced by LPC, then Krebs solution (**a**), DBT (**b**), ASR(**c**), AMR (**d**), Ferulic acid (**e**) and Calycosin-7-glucoside (**f**) to treat the rings. Each experiment were performed in triple. ^#^*p* < 0.05, ^##^*p* < 0.01, ^###^*p* < 0.001 when compared with the vehicle group; *p < 0.05, **p < 0.01, ***p < 0.001 when compared with the model group.

Rat aortic rings were incubated with Lysoph-Osphatidyl choline (LPC) (5 μg/mL) in Kebris solution for 30 min. Compared with normal group, the relaxant ratios of LPC group significantly decreased (p < 0.01) (Fig. 10a), suggesting that endothelium relaxant reaction of rings in LPC group were inhibited. Therefore, endothelial dysfunction model was successfully established.

#### Effects of DBT and its herbs on endothelial dysfunction

Rat aortic rings injured by LPC were treated with low, medium and high concentrations of decoctions. Results showed that DBT (Fig. 10b), ASR (Fig. 10c) and AMR (Fig. 10d) could relieve relaxant reaction of damaged rings in a dose-dependent manner, which suggested that DBT and its herbs could recover endothelial dysfunction induced by LPC in some degree.

#### Effects of ingredients on endothelial dysfunction

Rat aortic rings injured by LPC were treated with low, medium and high concentrations of ferulic acid and calycosin. Results showed that ferulic acid (Fig. 10 e) and calycosin-7-glucoside (Fig. 10f) could relieve relaxant reaction of rings in a dose-dependent manner. It suggested that these two pure compounds could recover endothelial dysfunction induced by LPC in some degree.

## Discussion

Until now, the compatibility mechanism of DBT for vascular diseases has remained unclear. Therefore, this work aimed to investigate the compatibility mechanism from the views of its chemical ingredients and effects on angiogenesis and endothelial function.

Quality of herbs was very important for pharmacologic functions. Herbs used in follow-up experiments were examined according to monographs of AMR and ASR in “European Pharmacopoeia” (Edition 8.0). The results showed that herbs in DBT satisfied the quality criteria and were suitable for the following experiments.

Then, we analyzed chemical ingredients in DBT using HPLC methods. As in published literature, calycosin and ferulic acid were indicated as effective ingredients^9^,^10^. In this paper four marker ingredients including calycosin, calycosin-7-glucoside and formononetin from AMR, and ferulic acid from ASR, were selected as marker compounds. Results showed that the contents of ferulic acid and calycosin-7-glucoside in DBT were markedly higher than the single herbs, suggesting that decocting process, in which AMR and ASR were extracted together, might promote the dissolution of these two aqueous ingredients. It was thus reasonable to conclude the compatibility of AMR and ASR in DBT. Furthermore, with the increasing of proportion ratio of AMR to ASR in DBT decoction, the synergistic scores were elevated. When ratio arrived in 5 to 1, the synergistic score was the best, which it demonstrated that the classic ratio (5:1) made senses.

As we all known, cardiovascular diseases often involved in angiogenesis disorder and endothelial dysfunction. Angiogenesis disorder might enhance endothelial cell dysfunction. Previous literatures had showed that DBT could promote the development of vascularization and accelerate proliferation of endothelial cells^11^,^12^. Therefore, we investigated compatibility of DBT from the two aspects.

For angiogenesis, the compatibility of DBT was studied using zebrafish model for the following reasons: The metabolism system of zebrafish was functionally similar to mammal^13^, both high-fecundity and rapid generation also supported zebrafish as a high-throughput screening model; some documents further confirmed that zebrafish results were consistent with clinical observations. Final results confirmed that both AMR and ASR could promote vessel growth. And these two herbs showed synergistic effect when used in combination, and the maximum effects were achieved when the ratio of AMR to ASR was classical (5:1). According to literatures, calycosin^14^, ferulic acid^15^ and polysaccharide fractions isolated from AMR^16^ might promote vascular growth. Therefore, in this paper, the effects of total extract and pure compounds on angiogenesis were evaluated respectively. Our results showed that AMR extract, AMRTS, ASRP, ferulic acid, calycosin and calycosin-7-glucoside had obvious angiogenesis effects on zebrafish model, indicating that these ingredients might be effective components in DBT. Therefore, we concluded that compatibility of DBT could enhance angiogenesis functions probably due to more extensive dissolution of effective ingredients.

For endothelial function, compatibility of DBT was studied using rat aortic rings which were pre-damaged by LPC. Results indicated that DBT as well as AMR and ASR herb extracts could relieve endothelial dysfunction. FA and calycosin-7-glucoside were also able to treat endothelial dysfunction, suggesting that these two components could be effective ingredients in DBT for endothelial function.

In summary, the chemical and pharmacologic results supported the compatibility of DBT formulae. When ASR and AMR were combined at the classical ratio (5:1), the dissolution of effective ingredients was increased, the angiogenesis effects were enhanced and the endothelial dysfunction was relieved.

## Methods and Materials

### Chemical analysis for DBT and its herbs

The quality of ASR herb and AMR herb were examined respectively according to the two monographs named as “angelica sinensis root” (07/2012:2558) and “astragalus mongholicus root” (01/2011:2435) in European Pharmacopoeia (8.0). Based on the conditions of single herbs, DBT formula was analyzed using HPLC method.

### Instruments and chromatographic conditions

For ASR, HPLC analysis of ASR was carried out using Thermo Scientific Dionex UltiMate 3000 system combined with Diode Array Detection (DAD) detection. Data was analyzed using Chromeleon (version 6.8). Chromatographic separations were performed with a Silice Upti-prep Strategy 100 A column (5 μm, 25 mm × 4.6 mm) at 25 °C. The mobile phases consisted of acetonitrile (A) and water (B) which contain 0.085% phosphoric acid (v/v). Analytes were separated with isocratic elution at the ratio of 13% A to 87% B with the flow rate of 1.0 ml/min. The Ultraviolet (UV) detection wavelength was set as 316 nm. The injection volume was 10 μl.

For AMR, HPLC analysis of AMR was carried out with a system of UPLC Acquity Waters with an Evaporative Light-Scattering Detector (ELSD) with air as the carrier gas (1.5 ml/min). Data was analyzed with logiciel Mass Lynx 4.1 version. Chromatographic separations were performed using a Waters acquity HSS T3 column (1.8 μm, 100 mm × 2.1 mm) at 40 °C with the flow rate of 0.6 ml/min. The temperature of drift tube was 40 °C. Injection volume was 2 μl. The mobile phases consisted of water (A) and acetonitrile (B), and in both phases 0.1% formic acid (v/v) was added. The gradient program was set as the following: 0~0.5 min, isocratic elution at 90.0% (A); 0.5~10 min, 90.0~37.0% (A); 10~13 min, 37.0~0.0% (A).

For DBT, HPLC was performed with the following operation parameters: column temperature was 35 °C; flow rate was 0.25 ml/min; 2 μl sample was filtered through millipore filter (0.22 μm) before injection; mobile phase consisted of 0.2% (v/v) phosphoric acid (A) and acetonitrile (B). For ferulic acid and calycosin-7-glucoside, the ratio of mobile phase (A to B) was 90 to 10, and UV signals were detected at 254 nm and 316 nm. For formononetin and calycosin, the ratio of mobile phase (A to B) was 75 to 25, and UV signals were detected at 254 nm and 316 nm.

### Preparation of sample solutions

0.20 g powder of ASR was heated and refluxed for 30 min with 20.0 ml mixture of methanol and water (7:3, v/v). Loss volume was replenished to 20 ml scale with same solution. Then extracting solution was mixed well, centrifuged at 10,000 r/min and filtrated through 0.45 μm membrane. Supernatant was obtained and stored at −4 °C before HPLC analysis.

4.0 g powder of AMR was accurately weighed into a Soxhlet extractor and soaked in 40 ml methanol overnight. In the next day, soften powder was replenished up to 40 ml with appropriate amount of methanol, heated and refluxed for 4 h. The methanol was recycled while the residue was dissolved in 10 ml water. Then the water solution of residue was extracted four times (40 ml each time) using butyl alcohol, which was pre-saturated with water. Next, the butyl alcohol layers of four times were mixed and the water layer was discarded. Then butyl alcohol solutions were completely washed twice with ammonia (40 ml per time). The ammonia layer was discarded, and the butyl alcohol layer was evaporated to dryness. The residue was dissolved in 5 ml water and loaded onto a solid phase extraction column containing 1 g octadecylsilyl silica gel and pre-washed with 5 ml methanol and 5 ml water. After loaded with drug, the column was sequentially eluted respectively with 20 ml water, 25 ml 25% alcohol and 70% alcohol. The elution of water and 25% alcohol was discarded, but 70% alcohol elution was kept and evaporated to dryness. Obtained residue was dissolved in appropriate amount of methanol, transfer into a 5 ml volumetric flask and replenished to 5 ml scale with methanol. The solution was mixed, centrifuged at 10,000 r/min and filtrated through 0.45 μm membrane. The supernatant was obtained and stored at 4 °C before HPLC analysis.

For single herbs, a portion of AMR (20 g) and ASR (20 g) were weighed. For the DBT, AMR (Lot: 110625) and ASR (Lot: 120119) were weighed according to the ratios of the formula. Herbs was soaked in the water for 90 min and decocted three times with 10 times water (20 min/time). The obtained decoctions were concentrated to 1 g/ml.

### Preparation of reference solutions

Solution (a):10.0 mg of ferulic acid was accurately weighted, transferred into 100 ml brown-glass volumetric flask, dissolved and diluted up to 100 ml with appropriate amount mixture of methanol and water at the volume ratio of 7 to 3. Solution (b): 2 ml of ferulic acid solution (a) was transferred into a transparent volumetric flask and exposed under 254 nm ultraviolet light for about 60 min, during which ferulic acid was transformed into cis-ferulic acid by UV light.

10.0 mg of astragaloside IV was dissolved in methanol and diluted to 10.0 ml scale with the same solvent. Then the reference (1 mg/ml) solution was obtained.

### Determination of ferulic acid and astragaloside IV

For ASR, content of ferulic acid was calculated using the following equation (1):


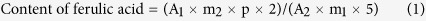


A_1_ = peak area of sampleA_2_ = peak area of reference solution (a);m_1_ = mass of the herbal drug (g)m_2_ = mass of pure compound (g)p = purity of ferulic acid (99.8%).

For AMR, content of astragaloside IV was calculated using the following equation (2):


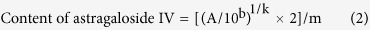


A = peak area of astragaloside IV;K = slope of calibration curveb = intercept of calibration curvem = mass of herbal drug (g).

Calibration curve of astragaloside IV was constructed by plotting peak area logarithm versus the concentration logarithm of the analyte.

For DBT sample, 1 ml of DBT and its single herbal solution (1 g/ml) were accurately pipetted, transferred into a 10 ml volumetric flask and diluted to 100 mg/ml with methanol. The mixtures were sonicated for 20 min, centrifuged at 10,000 rpm/min for 10 min and filtered through a 0.22 μm microporous membrane. All solutions were stored at −4 °C before analysis. The contents of the marked ingredients were calculated according to the calibration curves previously constructed. The normalized scores of the marked ingredients were obtained according to the following equation: normalized scores = (Content of sample − Min content)/(Max content − Min content) × 100%. The synthetic scores of four marked ingredients were calculated when the weight coefficient of each ingredient was one quarter.

### Method validation

For the standard solution, each accurately weighed standard was dissolved in methanol, and then diluted with methanol to different concentrations. Calibration curves were constructed by plotting the chromatographic peak area ratios versus the concentration of the analyte.

For the intra-day and inter-day precision of HPLC method, variability of the standard solutions were measured. The intra-day variation was tested with six replicates within one day and the inter-day variation was determined in duplicates for three consecutive days, and RSD was calculated.

For the repeatability test, means of three repetitive analyses for the same sample were evaluated in accordance with the recommended procedure^17^.

For the accuracy test, known amount of analytes were added into DBT and analyzed in triplicate for three consecutive days. The recovery was calculated by the following equation: recovery (%) = 100 × (found amount-original amount)/amount spiked.

For the stability of sample solution, the same sample of DBT was analyzed at 0, 2, 4, 24, and 48 h within 2 days under room temperature.

### Maintenance of zebrafish

All animal experiments were conducted in accordance with the ethical guidelines of the Institute of Chinese Medical Sciences and endorsed by the animal experimentation ethics committee of Shanghai University of Traditional Chinese Medicine.

The transgenic zebrafish lines with Tg (fli-1: EGFP) in which endothelial cells express EGFP were maintained as described in the zebrafish handbook^18^. In brief, it was maintained in a controlled environment at 28 °C on a 14 h: 10 h light/dark cycle (lights on at 08:00). Zebrafish were stored in 5 gallon tanks, continuously supplied with filtered reverse osmosis H_2_O. Fish were fed twice daily with brine shrimp in the morning and afternoon and also with general tropical fish food occasionally. In order to produce baby shrimp, 10 ml of shrimp eggs was added to 2 L of salt water without any antibiotics. After incubating the shrimp eggs for 48 h at 28.5 °C, the shrimps were filtered through a cloth, washed with fresh water and diluted into dH_2_O at a ratio of 1 volume shrimp to 3 volumes water. In order to feed zebrafish, 1 pasteur pipette of diluted shrimp was added for 8 adult fish. About half of the water was replaced to keep the environment healthier once a week.

### Embryo collection

Embryos were generated by natural pair-wise mating when the fish were 3~12 months old. The filters were switched off and breeding boxes were placed into the tanks, followed by exposure to light. The fish was left undisturbed for 15~30 min. Breeding boxes were collected and the embryos were transferred into clean petri-dishes with a fine fishing net and the embryos were maintained in Milli-Q water. Healthy, transparent and regular embryos were picked out at the 1~4 cell stage and were distributed into a 24-well microplate with 6~8 embryos per well depending on the assay. The morphological changes of embryos were observed using an Olympus Spinning Disk Confocal Microscope System (Nikon, Japan).

### Drug toxicity

At 20 hpf, target drugs (DBT, AMR, ASR, extracts and pure compounds) were separately added into microplates at different concentrations. At 48 and 72 hpf, toxicities of drugs to zebrafish including teratogenesis and lethality were observed. The tolerable concentrations were the maximum safety concentrations of the target drugs.

### Effects of drugs on SIVs of normal zebrafish

At 48 and 72 hpf, under the tolerable upper concentrations of target drugs, diameters of SIVs were measured and numbers of buds were recorded using Image J software (National Institute of Mental Health, USA). The effects of drugs on SIVs of normal zebrafish were evaluated.

### Effects of drugs on ISVs of zebrafish inhibited by VRI

15 nM VRI, which can inhibit the growth of ISVs, was added to the wells except the blank control and negative control groups with media containing target drugs at 24 hpf. After 48 h, the medium containing VRI was sucked out except the negative control group, washed three times with medium and respectively replaced with media containing target drugs.

The embryos receiving fresh media served as the vehicle control. At 24 and 48 hpf, intact and defective ISVs numbers in each zebrafish were counted. Intact ISV means the vessels from DA or PCV to DLAVs. In contrast, those vessels which elongate from DA or PCV but not connect DLAVs are defined as defective ISVs. Effects of drugs on ISVs of zebrafish inhibited by VRI were reflected using an index which was calce equation^19^:


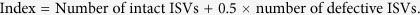


### Aortic ring preparation

All experimental procedures were performed according to the guidelines of the National Animal Welfare Law of China (ethics approval NO. SCXK2013-002). Sprague Dawley rats (200~250 g, bisexual each half) were purchased from Shanghai laboratory animals limited liability company (Shanghai, China). The animals were maintained at constant temperature (21~25 °C) and humidity (50~70%) on a 12 h/12 h light/dark cycle with free access to food and water. The animals were allowed to adapt to the laboratory environment for at least 14 days prior to the experiments.

Rats were sacrificed by cervical dislocation and exsanguinated. The thoracic aorta was dissected out, removed of adhering connective tissues, and cut into ring segments. Each ring was suspended between two stainless wire hooks in a 10 ml organ chambers filled with Krebs solution that contained (mM) (NaCl 119, KCl 4.7, MgCl_2_ • 6H_2_O 1, NaHCO_3_ 25, KH_2_PO_4_ 1.2, D-glucose 11.1, CaCl_2_ 2.5). The wire was fixed to the Tissue Organbath System (750TOBS, DMT). The bathing solution was oxygenated by 95% O_2_ and 5% CO_2_ and maintained at 37 °C (pH 7.4).

### Isometric force measurement

All rat aorta rings were placed under an optimal resting tension of 25 mN and equilibrated for 60 min. Next, rings were incubated with 60 mM KCl for 15 min to be depolarized. Then rings were rinsed triple with Krebs solution till they were completely relaxed. The depolarized process should be repeated in triple. Then rings were incubated with 1 μM phenylephrine for 15 min to be contracted. Their tension increased until the values equilibrated. Then accumulated 0.01~100 μM Ach were added into the rings, which were relaxed. Relaxant percentage of the rings was calculated according to the following equation: Relaxant percentage = relaxant values induced by Ach/maximal contractive value induced by phenylephrine. When relaxant percentage was more than 80%, the endothelium of this ring was considered as intact and suitable for future experiment.

### Experimental protocols of endothelial function

The qualified rings were randomly divided into three groups. In normal group, rings were incubated for 30 min with Krebs solution. In the model group, rings were incubated for 30 min with Krebs solution containing 5 mg/L LPC. In drug treatment groups, rings were treated with LPC and drugs. After rinsing twice with Krebs solution, all rings were contracted by 1 μM phenylephrine and relaxed by 0.01–100 μM Ach. The relaxant percentage was determined. Each experiment was performed in triple.

### Statistical analysis

Each experiment was repeated at least three times. Data were expressed as mean ± SEM. Statistical comparisons between groups were performed using one-way ANOVA followed by Dunnett’s t-test using non-treatment as the control group. *p* < 0.05 was considered statistically significant. SPSS 11.0 and Prism 6.0 software was used to analyze data and draw graph.

## Additional Information

**How to cite this article:** Lin, P.-L. *et al*. Compatibility study of Danggui Buxue Tang on Chemical ingredients, Angiogenesis and Endothelial Function. *Sci. Rep.* **7**, 45111; doi: 10.1038/srep45111 (2017).

**Publisher's note:** Springer Nature remains neutral with regard to jurisdictional claims in published maps and institutional affiliations.

## Figures and Tables

**Table 1:** The establishment of calibration curves for HPLC analysis.

Analyte	Calibration curve	R	Linear range (ng)
Ferulic acid	Y = 11273.44x + 2.03	0.9986	14.08~176.00
Calycosin-7-glucoside	Y = 11453.30x + 42.01	0.9982	16.64~166.40
Calycosin	Y = 8484.20x + 39.41	0.9975	26.40~88.00
Formononetin	Y = 3793.73x + 5.40	0.9986	12.48~208.00

**Table 2:** The recovery, LOD and LQD of pure compounds in DBT.

Analyte	Spiked (μg)	Found (μg)	RSD (%)	Recovery (%) (n = 3)	LOD (ng)	LOQ(ng)
Ferulic acid	71.88	68.23 ± 0.18	0.27	94.93		
	89.84	91.90 ± 0.33	0.36	102.29	3	14
	107.81	105.17 ± 0.66	0.51	97.4		
Calycosin-7-glucoside	72.96	75.56 ± 1.16	1.53	103.58		
	97.27	97.70 ± 2.84	2.91	100.43	3	9
	121.59	110.46 ± 0.23	0.21	90.84		
Calycosin	83.36	82.58 ± 2.51	3.04	99.06		
	116.71	122.33 ± 0.17	0.14	104.82	2	6
	150.06	155.24 ± 1.12	0.72	103.46		
Formononetin	144.9	140.86 ± 0.81	0.57	97.21		
	195.06	196.19 ± 0.53	0.27	100.58	5	15
	245.21	252.97 ± 6.82	2.7	103.16		

**Table 3:** The reproducibility of DBT.

	Ferulic Acid		Calycosin-7-glucoside		Calycosin		Formononetin	
	tR	A	tR	A	tR	A	tR	A
1	7.369	203.4	12.001	597.5	2.974	180.7	10.35	48.7
2	7.248	205.7	11.974	609	2.975	180.8	10.432	48.7
3	7.338	204.7	11.998	590.7	2.971	181.1	10.341	48.8
Average	7.32	204.60	11.99	599.07	2.97	180.87	10.37	48.73
SD	0.06	1.15	0.01	9.25	0.00	0.21	0.05	0.06
RSD(%)	0.86	0.56	0.12	1.54	0.07	0.12	0.48	0.12

**Table 4:** The stability test.

Time	Ferulic Acid		Calycosin-7-glucoside		Calycosin		Formononetin	
	tR	A	tR	A	tR	A	tR	A
0 h	7.369	203.4	12.001	597.5	2.974	180.7	10.35	48.7
2 h	7.369	203.4	12.001	597.5	2.974	180.7	10.35	48.7
4 h	7.248	205.7	11.974	609	2.975	180.8	10.432	48.7
24 h	7.338	204.7	11.998	590.7	2.971	181.1	10.341	48.8
48 h	7.359	199.2	12.018	595.7	2.952	180	10.285	49.2
Average	7.34	203.28	12.00	598.08	2.97	180.66	10.35	48.82
SD	0.05	2.48	0.02	6.71	0.01	0.40	0.05	0.22
RSD(%)	0.70	1.22	0.13	1.12	0.33	0.22	0.51	0.44

**Table 5:** The contents of major chemical ingredients presented in AMR, ASR and DBT.

AMR:ASR	Ferulic Acid (mg/g)	calycosin-7-glucoside (mg/g)	calycosin (mg/g)	formononetin (mg/g)
ASR	0.48	—	—	—
1:5	0.49	0.34	0.12	0.15
5:6	0.57	0.45	0.13	0.16
1:1	0.53	0.43	0.12	0.19
2:1	0.58	0.39	0.12	0.17
5:2	0.66	0.45	0.12	0.17
10:3	0.62	0.43	0.12	0.23
4:1	0.69	0.44	0.13	0.20
5:1	0.67	0.42	0.13	0.20
6:1	0.64	0.42	0.12	0.18
AMR	—	0.37	0.19	0.45

**Table 6:** The synthetic scores of the marker compounds in different ratios of DBT Decoction.

AR:ASR	AF	Calycosin-7-glucoside	Calycosin	Formononetin	Scores
1:5	0.00	0.00	0.00	0.00	0.00
5:6	38.10	100.00	91.61	12.69	60.60
1:1	21.56	76.08	26.57	56.22	45.11
2:1	44.24	38.94	33.57	27.86	36.15
5:2	84.78	98.20	36.36	32.21	62.89
10:3	63.41	76.08	32.17	100.00	67.92
8:2	100.00	84.44	83.92	60.82	82.29
5:1	89.66	69.24	100.00	66.42	81.33
6:1	73.12	69.33	14.69	40.55	49.42

**Table 7:** The maximum safety concentration of drugs at 48 hpf.

Drug	Maximum safety concentration (μM)	Drug	Maximum safety concentration (μg/ml)
Calycosin	30	DBT	250
Calycosin-7-glucoside	30	AR	250
Formononetin	100	ASR	250
Astragaloside IV	30	AR extract	100
Ferulic Acid	10	ARTS	100
		ARP	100
		ASR extract	30
		ASRP	100
