# Author Correction: Compatibility study of Danggui Buxue Tang on Chemical Ingredients, Angiogenesis and Endothelial Function

**DOI:** 10.1038/s41598-022-06353-x

**Published:** 2022-02-03

**Authors:** Ping-Lan Lin, Zhi-Cheng Li, Rui-Fang Xie, You-Hua Wang, Xin Zhou

**Affiliations:** 1 Department of Pharmacy, Longhua Hospital Affiliated to Shanghai University of Traditional Chinese Medicine, Shanghai, China; 2 Department of Nephrology, Shuguang Hospital Affiliated to Shanghai University of Traditional Chinese Medicine, Shanghai, China; 3 Shanghai Pu Dong Hospital Affiliated to FuDan University, Shanghai, China; 4 Hypertension Lab, Cardiovascular Department, Longhua Hospital Affiliated to Shanghai University of Traditional Chinese Medicine, Shanghai, China

Correction to: *Scientific Reports* 10.1038/srep45111, published online 22 March 2017

Some of the data in this Article has been previously published in a paper which reported preliminary results from this study^1^.

Figure 2 in this Article is based on the same data as Figure 1 in^1^.

Panels 5A-D and 6A-D (which show the same data) in this Article were shown in panels 2A-D in Figure 2 in^1^.

Calibration data shown in Table 1 in this Article was reported in Table 1 in^1^.

Contents of DBT's main ingredients reports in Table 5 in this Article were also reported in Table 2 in^1^.

Results reported in Table 6 in this Article were reported in Table 3 in^1^.
